# Mapping splice QTLs reveals distinct transcriptional and post-transcriptional regulatory variation of gene expression and identifies putative alternative splicing variation mediating complex trait variation in pigs

**DOI:** 10.1186/s12864-023-09314-4

**Published:** 2023-05-05

**Authors:** Fei Zhang, Deborah Velez-Irizarry, Catherine W. Ernst, Wen Huang

**Affiliations:** Department of Animal Science, Michigan State University, East Lansing, MI 48824 USA

**Keywords:** Splice QTL, Expression QTL, Regulatory variation of gene expression, Pigs

## Abstract

**Background:**

Alternative splicing is an important step in gene expression, generating multiple isoforms for the same genes and greatly expanding the diversity of proteomes. Genetic variation in alternative splicing contributes to phenotypic diversity in natural populations. However, the genetic basis of variation in alternative splicing in livestock including pigs remains poorly understood.

**Results:**

In this study, using a Duroc x Pietrain F2 pig population, we performed genome-wide analysis of alternative splicing estimated from stranded RNA-Seq data in skeletal muscle. We characterized the genetic architecture of alternative splicing and compared its basic features with those of overall gene expression. We detected a large number of novel alternative splicing events that were not previously annotated. We found heritability of quantitative alternative splicing scores (percent spliced in or PSI) to be lower than that of overall gene expression. In addition, heritabilities showed little correlation between alternative splicing and overall gene expression. We mapped expression QTLs (eQTLs) and splice QTLs (sQTLs) and found them to be largely non-overlapping. Finally, we integrated sQTL mapping with phenotype QTL (pQTL mapping to identify potential mediator of pQTL effect by alternative splicing.

**Conclusions:**

Our results suggest that regulatory variation exists at multiple levels and that their genetic controls are distinct, offering opportunities for genetic improvement.

**Supplementary Information:**

The online version contains supplementary material available at 10.1186/s12864-023-09314-4.

## Background

Expression of genes is regulated by a multitude of molecular processes which collectively determine the abundance and spatiotemporal distribution of gene expression products of different forms. Both genetic and environmental factors can lead to variation in gene expression regulation, which can ultimately cause variation in organismal phenotypes within and between species. For example, it has been well established that variation in gene expression contributes to the divergence between species [1]. Genome-wide mapping efforts have identified numerous expression quantitative trait loci (eQTLs), i.e. DNA variants that are associated with changes in gene expression, within different organisms, including plants [2], humans [3], insects [4], and livestock [5].

Alternative splicing of pre-mRNA is a major step in post-transcriptional regulation of complex eukaryotic gene expression. The most prominent consequence of alternative splicing is the vast expansion of the transcriptome and proteome by forming multiple transcript isoforms, which have the potential to code for different proteins [6]. Alternative splicing is tightly controlled, often in a cell type and developmental stage specific manner, to regulate fundamental biological processes including growth and development. Despite the importance of alternative splicing in gene expression regulation, the genetic basis of alternative splicing variation between and within species and its relationship with phenotypic variation remain less understood than steady state RNA abundances.

Many non-genetic and factors can influence alternative splicing, including developmental stages [7], cell and tissue types [8], disease status [9], as well as sequence variation between individuals [10]. Importantly, genetic variation in alternative splicing is abundant and contributes significantly to phenotypic variation in natural populations [10]. For example, aberrant alternative splicing caused by mutations in *cis* regulatory elements or splicing factors underlies many human diseases [11–13]. In livestock, QTLs affecting alternative splicing have been mapped in dairy and beef cattle and found to be extensively shared across tissues [14]. Many tissue specific QTLs influencing alternative splicing were also present [14]. In addition, eQTLs and splice QTLs (sQTLs) were found to be associated with meat quality traits in a population of Angus-Brahman crossbred cattle [15].

In a previous study, we mapped hundreds of eQTLs for RNA abundance in the *longissimus dorsi* muscle of pigs from a Duroc x Pietrain F2 population, providing potential links to facilitate the functional interpretation and dissection of phenotype QTLs [5]. In the present study, we extended this work to map sQTLs, taking advantage of the paired-end and strand-specific RNA-Seq data. We compared the heritability of overall gene expression and alternative splicing which showed little correlation. Furthermore, the comparison of mapped eQTLs and sQTLs showed little overlap and different location patterns, suggesting distinct transcriptional and post-transcriptional regulatory variation of gene expression. Finally, we integrated sQTLs with mapped phenotype QTLs (pQTLs) to identify putative causal genes whose genetic variation in alternative splicing may have mediated the pQTL effects.

## Results

### Identification of alternative splicing events in pigs using RNA-Seq

To understand the genetic basis of alternative splicing regulation in skeletal muscle in pigs, we analyzed the RNA-Seq dataset from a previous study [5] to obtain both overall gene expression and alternative splicing quantifications in *longissimus dorsi* muscle in the MSU Pig Resource Population derived from a F2 cross of Duroc x Pietrain pigs. We focused on 143 of the 168 animals for which strand-specific RNA-Seq libraries were sequenced. On average, 45.8 million 125 bp paired-end reads were sequenced per sample, 37.5 million of which (Additional File 1, Table S1) were uniquely mapped using HISAT2 [16]. Importantly, of the uniquely mapped reads, 21.5 million covered junction sites, enabling us to classify and quantify alternative splicing (Table S1).

**Fig. 1:**
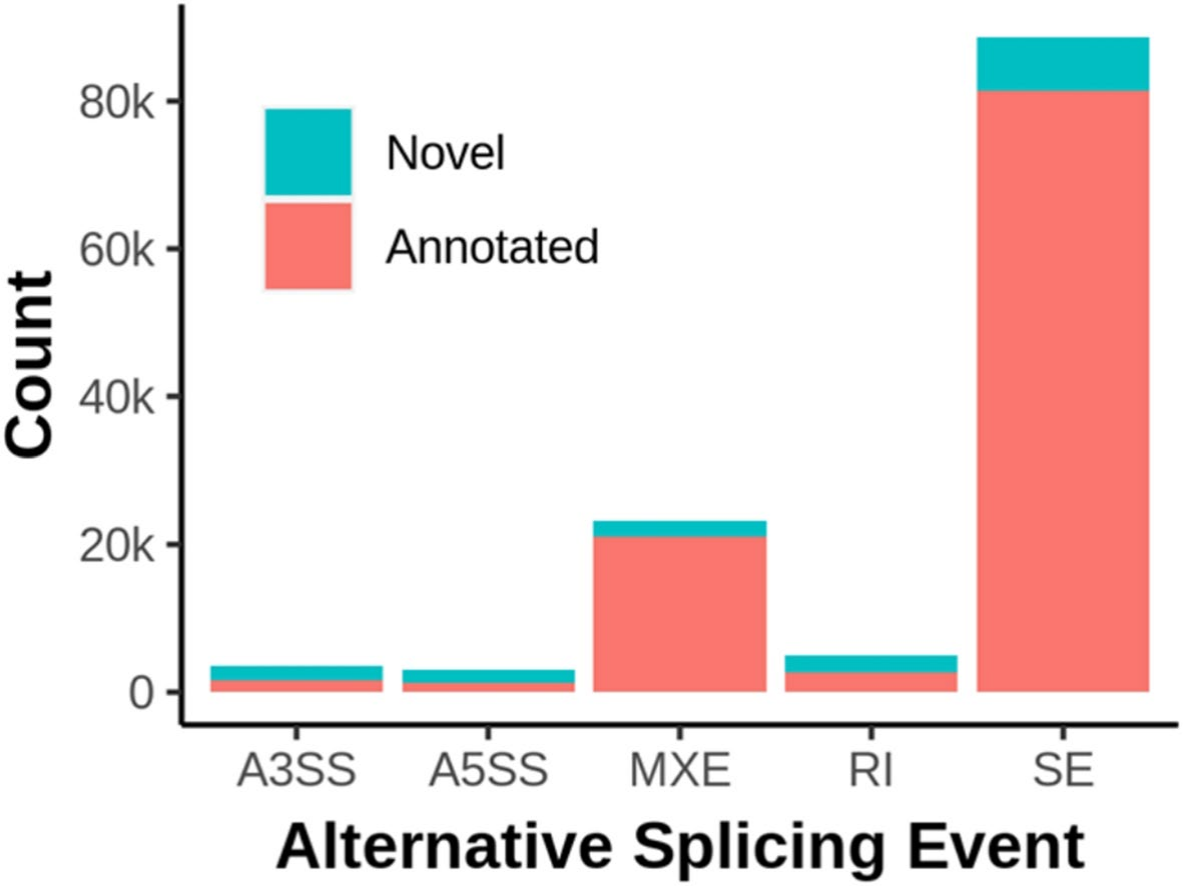
Identification of annotated and novel alternative splicing events in pig muscle by RNA-Seq. Stacked bar chart showing the counts of annotated and novel alternative splicing events supported by experimental evidence. We consider an event experimentally supported if both isoforms are supported by at least two reads in at least two samples. A3SS = alternative 3’ splice site; A5SS = alternative 5’ splice site; MXE = mutually exclusive exons; RI = retained intron; SE = skipped exon

We assembled transcript models from each sample using StringTie [17] and merged and filtered them to produce a combined annotation. We identified alternative splicing events from merged transcript annotations across all RNA-Seq samples using rMATs [18], which classified them into five classes, including skipped exon (SE), retained intron (RI), alternative 5′ splice site (A5SS), alternative 3′ splice site (A3SS), and mutually exclusive exons (MXE). We considered an alternative splicing event supported by experimental evidence in our muscle RNA-Seq data if both splice isoforms were supported by at least two junction reads in at least two samples. There were 88,670, 4,980, 3,044, 3,591, and 23,187 SE, RI, A5SS, A3SS, and MXE events respectively, among which, 7,323, 2,285, 1,720, 1,963, and 2,081 were novel (Fig. 1). Despite that only muscle RNA was sequenced, the large number of unannotated alternative splicing events testified to the power of population-scale RNA-Seq and the incompleteness of the current gene annotation in pigs. However, it should be noted that novelty of alternative splicing events in RNA-Seq data depends on completeness of reference annotations, which improves as large-scale genome annotation projects such as the FAANG accumulate data [19].

### Quantitative genetics of alternative splicing and steady state RNA abundance

To understand the genetic basis of alternative splicing and steady state RNA abundance, we first estimated the genomic heritability of these two types of traits. Genomic heritability quantifies the proportion of variation in alternative splicing or overall gene expression that can be attributed to additive genetic variance. We defined the percent spliced-in (PSI) score as the proportion of junction reads supporting one of the splice isoforms versus the sum of both isoforms to quantitatively represent alternative splicing.

We applied several non-specific filters to PSI and TPM (transcripts per million) representing overall gene expression, including filters on overall magnitude of variances, coefficient of variation and skewness of distribution. The remaining genes or alternative splicing events were Normal quantile transformed and fitted in a linear mixed model with genomic relationship matrix calculated from 50K SNP chip genotypes of the animals. Significance of the deviation of additive genetic variance from zero was determined using a likelihood ratio test comparing the full mixed model and a reduced model without the additive genetic variance component. At a false discovery rate (FDR) = 0.01, we identified 2,200 genes out of a total of 11,368 expressed genes that had significant heritability. For alternative splicing, 405 alternative splicing events in 190 genes were significant out of a total of 22,018 events in 6,004 genes. A total of 65 genes had both significant heritable overall gene expression and alternative splicing.

**Fig. 2:**
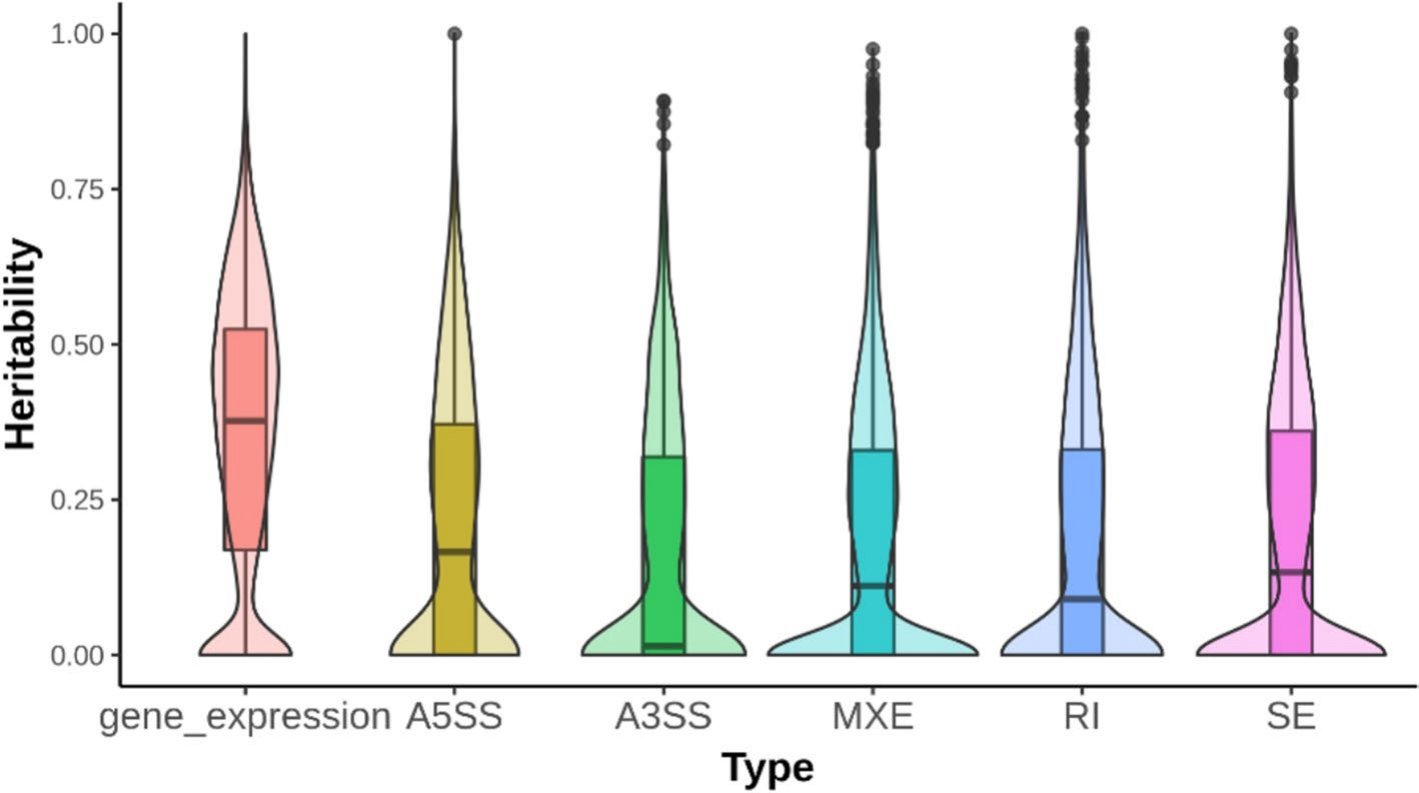
Distribution of genomic heritabilities for gene expression and alternative splicing traits. Violin plots overlaid on boxplots showing the distribution of genomic heritability for overall gene expression and five classes of alternative splicing. A3SS = alternative 3’ splice site; A5SS = alternative 5’ splice site; MXE = mutually exclusive exons; RI = retained intron; SE = skipped exon

**Fig. 3:**
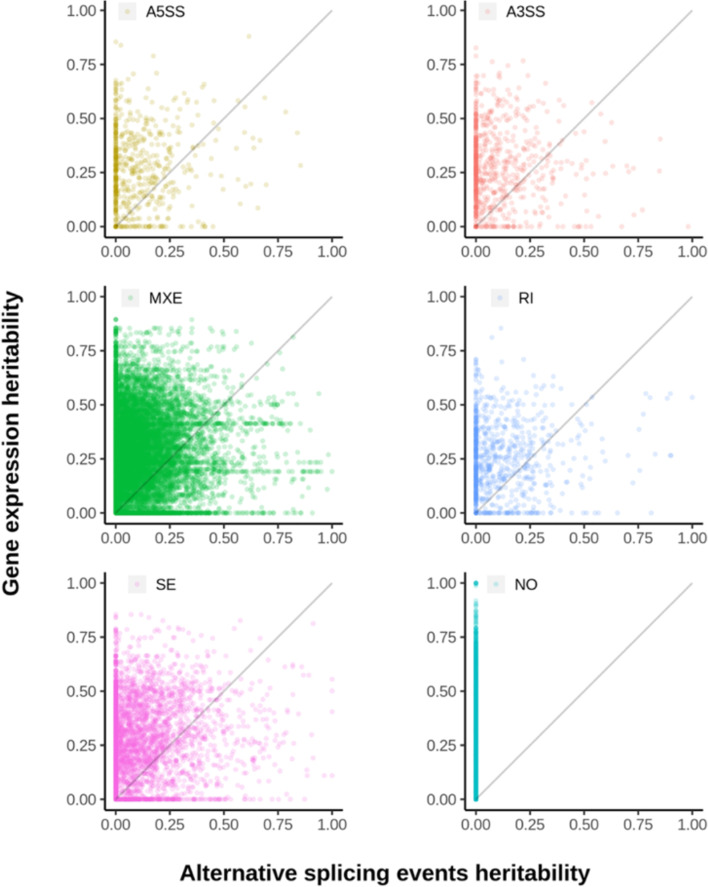
Comparison of heritability for alternative splicing and overall gene expression for the same genes. Scatter plots showing comparison between heritability for five classes of alternative splicing and overall gene expression. The diagonal line indicates perfect agreement. A5SS = alternative 5’ splice site; A3SS = alternative 3’ splice site; MXE = mutually exclusive exons; RI = retained intron; SE = skipped exon; NO = no alternative splicing events detected. Multiple events may exist for a gene and are represented by different points on the plots

The estimated heritability was in general larger for overall gene expression than for all five types of alternative splicing (Fig. 2). Importantly, the heritability of these two aspects of gene expression showed little correlation (Fig. 3). Bivariate quantitative genetic analysis suggested that the genetic correlation was generally small. Of the 20,625 gene expression and alternative splicing pairs, only 585 had a significant (FDR = 0.01) genetic correlation. These results suggested that the heritable genetic control of transcriptional and post-transcriptional regulation of gene expression may be distinct, even though the two molecular processes are highly coupled spatially, temporally, and functionally [20].

### Mapping splice QTLs (sQTLs) and expression QTLs (eQTLs)

**Fig. 4:**
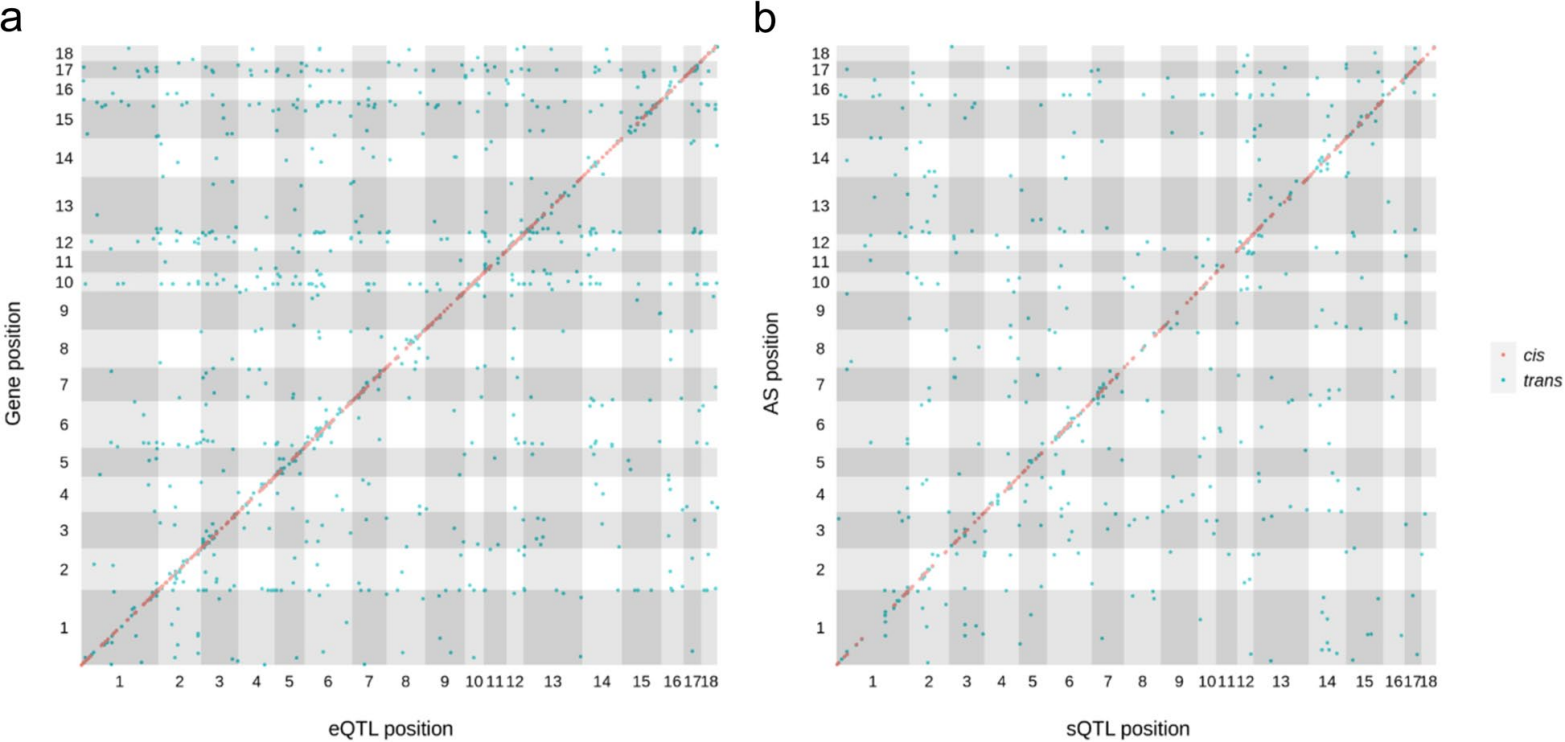
Locations of eQTLs and sQTLs relative to genes. eQTL (**a**) and sQTL (**b**) location plots showing the relative positions of eQTLs and sQTLs versus genes and alternative splicing events. Red and green dots indicate cis and trans QTLs respectively. Chromosomes are shown using alternative grey and white colors

To understand the distinct genetic control of transcription and splicing, we mapped splice QTLs (sQTLs) and expression QTLs (eQTLs) for all genes using genome-wide association analysis. At an empirical FDR = 0.01, we identified a total of 35,687 sQTLs for 796 alternative splicing events in 475 genes and 43,432 eQTLs for 1,098 genes (Fig. 4a, b). The large number of sQTLs and eQTLs per alternative splicing or gene expression trait was the result of the high level of linkage among genetic markers in the F2 population. We therefore performed a forward model selection approach to eliminate associations due to linkage and classified sQTLs and eQTLs based on the markers retained in the models. Both sQTLs and eQTLs exhibited an enrichment of *cis* (defined as 1Mb within the boundaries of genes) QTLs as compared to *trans*. Despite that *trans* QTLs have a much larger space to exist, of the 1,338 genes with at least one eQTL and 961 alternative splicing events with at least one sQTL, 552 (41%) and 467 (48%) had *cis* eQTLs and sQTLs, respectively (Table 1). These results, as many studies have shown for eQTLs previously, indicated that proximal regulation is a primary mode of regulation for both transcription and alternative splicing.

**Table 1:** Counts of eQTLs and sQTLs classified into *cis* and *trans*

	Total	*cis*	*trans*	both
Genes	1,338	552	865	79
Alternative splicing events	961	467	549	55

Among the 1,338 genes with eQTLs and 586 genes with sQTLs, 105 were in common. Of the 105 genes with mapped eQTLs and sQTLs, only 16 contained SNPs that were associated with both overall gene expression and alternative splicing. These results, together with the lack of correlation between SNP based heritability estimates for overall gene expression and alternative splicing and low genetic correlation, suggested that the genetic control of transcription and alternative splicing were likely distinct.

### Identification of alternative splicing variation that putatively mediates QTL effects on economic traits

**Fig. 5:**
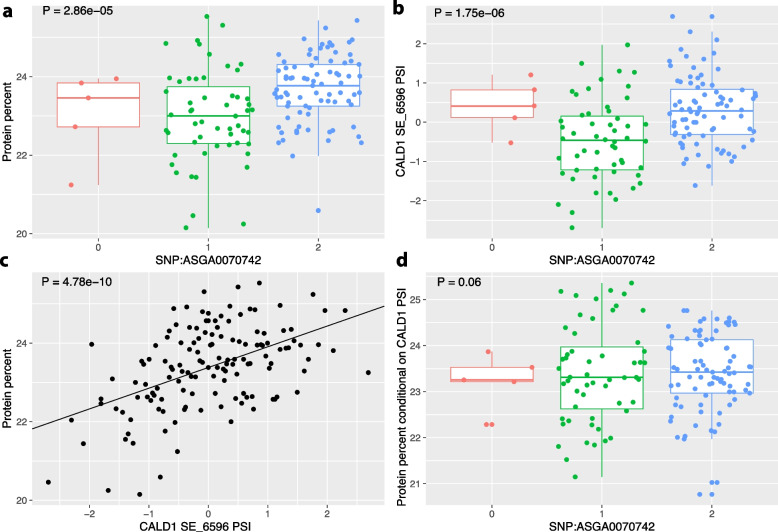
Mediation of pQTL effect by sQTL. **a** Effect of a pQTL SNP on the protein percent trait. **b** Effect of the same SNP is also a sQTL and its effect on the PSI of a skipped exon (SE) event in the gene CALD1. **c** Correlation between CALD1 PSI and protein percent. **d** Effect of the pQTL after adjusting for the CALD1 PSI

To understand the effects of sQTLs on economic traits and their distinction with eQTL effects, we performed GWAS to identify phenotype-associated QTLs (pQTLs) in this pig population for 67 economic traits [5]. Using a permutation based FDR threshold of 0.05, we identified 208 pQTLs for 17 unique traits. Similar to eQTL and sQTLs, pQTLs were clustered due to high level of LD in the F2 population. Many of these pQTLs are also sQTLs or eQTLs. Among the pQTLs, we identified a total of 389 trios of sQTLs, alternative splicing events (PSI), and economic traits, as well as 1,070 trios of eQTLs, genes, and economic traits. This allowed us to test for mediation of the pQTL effects on economic traits by alternative splicing and gene expression. We performed two tests for this mediation analysis. First, we asked if the association between the pQTL and the trait diminished when PSI or gene expression when PSI or gene expression was included as a covariate. Reduction in association would indicate that the pQTL effect was at least in part mediated by the alternative splicing or gene expression. Second, we tested for the proportion of mediation using a model based approach implemented in the mediation R package [21]. At an FDR = 0.05, we identified 15 sQTLs (Table S2) and 152 eQTLs (Table S3) that mediated pQTL effect on economic traits. For example, we identified a significant pQTL (ASGA0070742) for the protein percent in the muscle (Fig. 5a, *P* = 2.86e-5). The same pQTL is also a sQTL (Fig. 5b, *P* = 1.75e-6) for a skipped exon (SE) event in the caldesmon 1 (CALD1) gene, which plays an important role in muscle contraction. Remarkably, the PSI of the SE event in CALD1 is highly associated with protein percent (Fig. 5c, *P* = 4.78e-10). However, adjusting for CALD1 PSI nearly completely abolished the pQTL effect (Fig. 5d, *P* = 0.06), strongly suggesting that the pQTL effect was mediated by the alternative splicing in CALD1. Taken together, these results suggest that in addition to eQTLs [5], sQTLs can also provide important information regarding the molecular mechanism of how regulatory variation contribute to phenotypic variation.

## Discussion

In this study, we carried out an analysis to estimate heritability of alternative splicing and map sQTLs in skeletal muscle of 143 pigs in a Duroc x Pietrain F2 population [5]. The samples were deeply sequenced with stranded paired-end reads, enabling us to estimate both alternative splicing and steady state RNA abundances. While many studies have mapped eQTLs for overall gene expression in livestock, genetic characterization of alternative splicing remains understudied.

With a single tissue type, we found a significant number of novel alternative splicing events supported by multiple reads in multiple samples (Fig. 1). Skeletal muscle is one of the most intensively studied tissues in pigs and yet we were able to substantially improve existing annotations, underscoring the incompleteness of the present annotation, which is expected to further improve as large-scale annotation projects are completed [19].

By comparing genomic heritability of quantitative gene expression and alternative splicing, we found heritability of alternative splicing was generally lower than that of overall expression (Fig. 2). It’s important to point out that the magnitude of heritability depends on the precision of measurements. An explanation for the lower heritability for alternative splicing may be that alternative splicing utilizes only reads spanning junctions, whereas overall gene expression takes advantage of all exonic reads.

We found little correlation between the heritabilities of overall gene expression and alternative splicing (Fig. 3). This suggested that genetic controls of transcription and alternative splicing were likely distinct. Heritabilities for overall gene expression were generally higher than alternative splicing. There can be several reasons, including but not limited to higher environmental (non-genetic) sensitivity of alternative splicing and the necessity to use junction reads thus smaller coverage for PSI estimation. In addition, we estimated generally low genetic correlation between overall expression and alternative splicing with high standard errors. Out of 20,625 gene expression and alternative splicing pairs, only 585 had a significant (FDR = 0.01) genetic correlation. Both eQTLs and sQTLs were enriched for SNPs proximal to the genes they regulated, where *cis* regulatory elements were concentrated. A substantial fraction of genes contained both mapped eQTLs and sQTLs, yet only a small fraction shared the same genetic markers as eQTLs and sQTLs. The resolution of the F2 design and the density of the SNP chip did not allow us to perform a comprehensive evaluation of overlap. Taken together, these results suggested that transcription and alternative splicing were distinctly regulated genetically.

Ultimately, regulatory variation in transcription and splicing lead to phenotypic variation. We performed mediation analyses to identify alternative splicing events whose genetic variation may mediate the effects of pQTLs on phenotypic variation. Our results suggested that in addition to RNA abundance, alternative splicing can also facilitate interpretation of genetic associations between pQTLs and economic traits and identification of putative causal mediating factors. These efforts ultimately enhance genetic improvement of animals. Our study suggests that when considering regulatory variation as an intermediate step that connects DNA variation and organismal phenotypic variation, alternative splicing should not be ignored. Indeed, studies in humans suggest that alternative splicing may be a major contributor to phenotypic variation [10]. As large-scale annotation projects improve annotation of the pig genome, estimation of alternative splicing will become easier and more accurate, which will ultimately improve our ability to understand and utilize its contribution to phenotypic variation for genetic improvement of animals.

## Conclusions

Using a stranded RNA-Seq dataset in a F2 population of pigs, we mapped eQTL and sQTL in the skeletal muscle. We found overall gene expression and alternative splicing to have low genetic correlation, and heritabilities of these two classes of traits to have low correlation. In addition, although both classes of traits had QTLs concentrated near the genes, we found very little overlap between sQTLs and eQTLs. These results suggested that the genetic regulation of transcription and alternative splicing were likely distinct that both need to be considered when mapping regulatory variation that connects DNA variation and phenotypic variation.

## Materials and methods

### RNA-Seq data mapping and alternative splicing event calling

All RNA-Seq data were obtained from a previously published study with accession number PRJNA403969 [5]. To reduce the influence of batch effect, we focused on 144 samples which were prepared uniformly to produced stranded sequencing libraires and sequenced by the Illumina HiSeq 2500 platform with 125 bp paired end reads. These 144 F2 individuals were offspring of 35 F1 animals descended from 19 purebred animals. Raw sequence reads were aligned to the *Sus scrofa* reference genome (11.1) and transcriptome (Ensembl Release 97) using HISAT2 2.1.0 [16]. There were on average 45.8 million of reads per sample, with 90.9% overall mapping and 81.9% unique mapping rates. One of the samples (1833) had an uncharacteristically low (49.64%) unique mapping rate and was removed from further analysis, resulting in 143 samples. Mapped reads that covered junctions comprised 46.9% of all reads on average, providing a strong basis to estimate alternative splicing accurately. StringTie 2.04 [17] was used to assemble transcripts based on HISAT2 alignments, producing a gene annotation per sample. The StringTie parameters were set to allow a minimum transcript length of 200 bp, minimum transcript coverage of 2.5 and minimum isoform fraction of 0.1. Annotations from all samples were merged to a set of 78,351 transcripts, among which 29,193 (37.26%) were novel. Alternative splicing events were identified by rMATs 4.1.0 [18] using the combined gene annotations and novel alternative splicing was called if they did not exist in the reference annotation. rMATs identifies alternative splicing events by considering assembled transcript models and classifies them into five major categories including alternative 5’ splice site (A5SS), alternative 3’ splice site (A3SS), mutually exclusive exons (MXE), retained intron (RI), and skipped exon (SE). We conservatively considered novel events where both isoforms were supported by at least two reads in at least two samples.

### Heritability estimation of alternative splicing and gene expression

To quantify alternative splicing, percent splice-in (PSI) scores [18] were calculated for each gene using the following formula:

$$percent\;splice\;in\left(PSI\right)=\frac{inclusion\_counts/inclusion\_length}{inclusion\_counts/inclusion\_length+exclusion\_counts/exclusion\_length}$$

When estimating heritability, we only considered alternative splicing events with at least 5 total reads supporting both isoforms in at least 50% of the samples. Only alternative splicing events with a mean absolute deviation greater than 1, log2 variance greater than 2, skewness < 1.1 were considered variable and retained. In addition, to eliminate the influence of sex specific alternative splicing, we only retained events where samples from either sex contributed at least 20% of the estimable samples. PSI was normal quantile transformed using the R package bestNormalize [22]. The transformed PSI was used to estimate genomic heritability (*h*^*2*^) using the R package rrBLUP [23] with sex of the animal as a fixed effect. The R package fdrtool was then used to calculate the FDR value based on *P* values obtained from likelihood ratio tests.

Overall gene expression was estimated using transcript per million (TPM) output by StringTie. TPM of each gene was also subjected to a normal quantile transformation. The transformed TPM was then used to estimate the genomic heritability (h^2^) of gene expression by rrBLUP package. Genetic correlation was computed using the GCTA package [24]. The R package fdrtool was then used to calculate the FDR values based on the Benjamini-Hochberg approach.

### Expression QTL and splice QTL mapping

eQTL and sQTL mapping was performed using the fastGWA function of GCTA [24] with sex of the animal fitted as a covariate. To estimate empirical FDR, we permuted the data set (animal ID) 200 times while preserving correlation among genes and performed eQTL and sQTL mapping in the permuted datasets. Empirical FDR for each was estimated as the observed number of significant QTLs at a given threshold divided by the average number of significant QTLs among the 200 permutations. eQTLs or sQTLs located within 1 Mb of gene boundaries were consider *cis*, and otherwise *trans*.

### Mediation analysis

To identify potential mediator of pQTL effects, we first performed pQTL mapping in 67 traits using the same procedure as eQTL and sQTL mapping as described above. We then integrated pQTL, sQTL/eQTL, PSI/gene expression, and phenotypes using two approaches. First we tested association between pQTLs and phenotypes by adjusting the phenotypes for PSI or gene expression. Second, we performed a model based mediation analysis [21] to estimate proportion of the pQTL effect on phenotype that is mediated by the PSI/gene expression mediator. Significant mediators were identified at FDR = 0.05.

## Supplementary Information

**Additional file 1.**

## Data Availability

All Data are downloaded from a previous study with the accession number PRJNA403969 [5] from SRA. Codes are available at: https://github.com/zhangfei1947/MSUPRP.
